# Transdural Revascularization by Multiple Burrhole After Erythropoietin in Stroke Patients With Cerebral Hypoperfusion: A Randomized Controlled Trial

**DOI:** 10.1161/STROKEAHA.122.038650

**Published:** 2022-05-17

**Authors:** Ji Man Hong, Mun Hee Choi, Geun Hwa Park, Hee Sun Shin, Seong-Joon Lee, Jin Soo Lee, Yong Cheol Lim

**Affiliations:** Department of Neurology (J.M.H., M.H.C., G.H.P., S.-J.L., J.S.L), Ajou University School of Medicine, Ajou University Medical Center, Suwon, South Korea.; Department of Neurosurgery (Y.C.L.), Ajou University School of Medicine, Ajou University Medical Center, Suwon, South Korea.; Department of Biomedical Sciences, Ajou University Graduate School of Medicine, Suwon, South Korea (H.S.S.).

**Keywords:** cerebral angiography, cerebral infarction, demography, erythropoietin, perfusion

## Abstract

In patients with acute symptomatic stroke, reinforcement of transdural angiogenesis using multiple burr hole (MBH) procedures after EPO (erythropoietin) treatment has rarely been addressed. We aimed to investigate the efficacy and safety of cranial MBH procedures under local anesthesia for augmenting transdural revascularization after EPO treatment in patients with stroke with perfusion impairments.

**Methods:**

This prospective, randomized, blinded-end point trial recruited patients with acute ischemic stroke with a perfusion impairment of grade ≥2 within 14 days of symptom onset, steno-occlusive mechanisms on imaging examinations, and absence of transdural collaterals on transfemoral cerebral angiography. Patients were randomly assigned to receive MBH + EPO or MBH alone. The primary and secondary outcomes were revascularization success (trans-hemispheric and trans-burr hole) at 6 months and adverse events, respectively.

**Results:**

We evaluated 42 of the 44 targeted patients, with 2 patients lost to follow-up. The combined and MBH-only (n=21 each) groups showed no differences in demographic characteristics and baseline perfusion parameters. Significantly, more cases of trans-hemispheric (19/21 [90.5%] versus 12/21 [57.1%]) and trans-burr hole (42/58 [72.4%] versus 30/58 [51.7%]) revascularization and significant improvements in perfusion parameters were observed in the combined group relative to the MBH-only group. No differences in treatment-related complications were observed between groups. Even after adjustment for potential covariates, EPO usage was an independent factor of successful hemispheric revascularization in this study (odds ratio, 6.41 [95% CI, 1.08–38.02]).

**Conclusions:**

The combination of MBH and EPO is safe and feasible for reinforcing transdural revascularization in acute steno-occlusive patients with perfusion impairments.

**Registration:**

URL: https://www.clinicaltrials.gov; Unique identifier: NCT02603406.

Modern bypass cerebrovascular surgical techniques for ischemic tissue revascularization have mainly focused on the immediate anastomosis with a reverse transdural mode between the extracranial and the intracranial carotid systems.^1,2^ Such transdural collaterals offer one of the most protective vascular collaterals against impending cerebral infarction in cases of moyamoya disease or proximal vessel occlusion with intracranial perfusion impairment.^2,3^ However, in the acute period of neurologically unstable stroke with perfusion impairments, the aforementioned extensive bypass surgeries may not be preferable due to the considerable risk of complications during or after operations performed under general anesthesia.^4,5^ In some clinical studies on minimally invasive procedures, cranial multiple burr hole (MBH) procedures have been shown to be beneficial in certain cases, such as moyamoya disease, which is a progressive cerebrovascular occlusive disease affecting the circle of Willis.^6,7^ However, although the risk of complications arising from such MBH procedures is relatively low, a robust transdural anastomosis is not guaranteed^6,8^; the use of these procedures has thus been limited to a reliable angiogenesis modality for transdural revascularization in real-world situations.

A recent retrospective case series outlining the efficacy and safety of transdural revascularization reported positive results (≈98% successful revascularization without serious complications) with cranial MBH and systemic EPO (erythropoietin) pretreatment.^9^ EPO may be useful for promoting transdural revascularization because it can harness the angiogenic potential of endothelial cells and has also been proven to be safe in high-dose regimens in preclinical and human experiments.^10–14^ Therefore, we hypothesized that the combination of cranial MBH procedures with an EPO pretreatment regimen would be effective and safe for robust transdural revascularization even in patients with acute stroke with unstable clinical presentations due to intracranial perfusion deficits. To evaluate this hypothesis, this trial aimed to compare the efficacy and safety of cranial MBH procedures with or without intravenous EPO pretreatment for transdural revascularization in patients with acute symptomatic stroke (<2 weeks) showing perfusion impairment.

## Methods

### Data Availability

Anonymized data are available to qualified investigators at reasonable request by the corresponding author. This trial was completed in accordance with the CONSORT guidelines^15^; the CONSORT guidelines checklist is available in the Supplemental Material.

### Design

**Figure 1.**
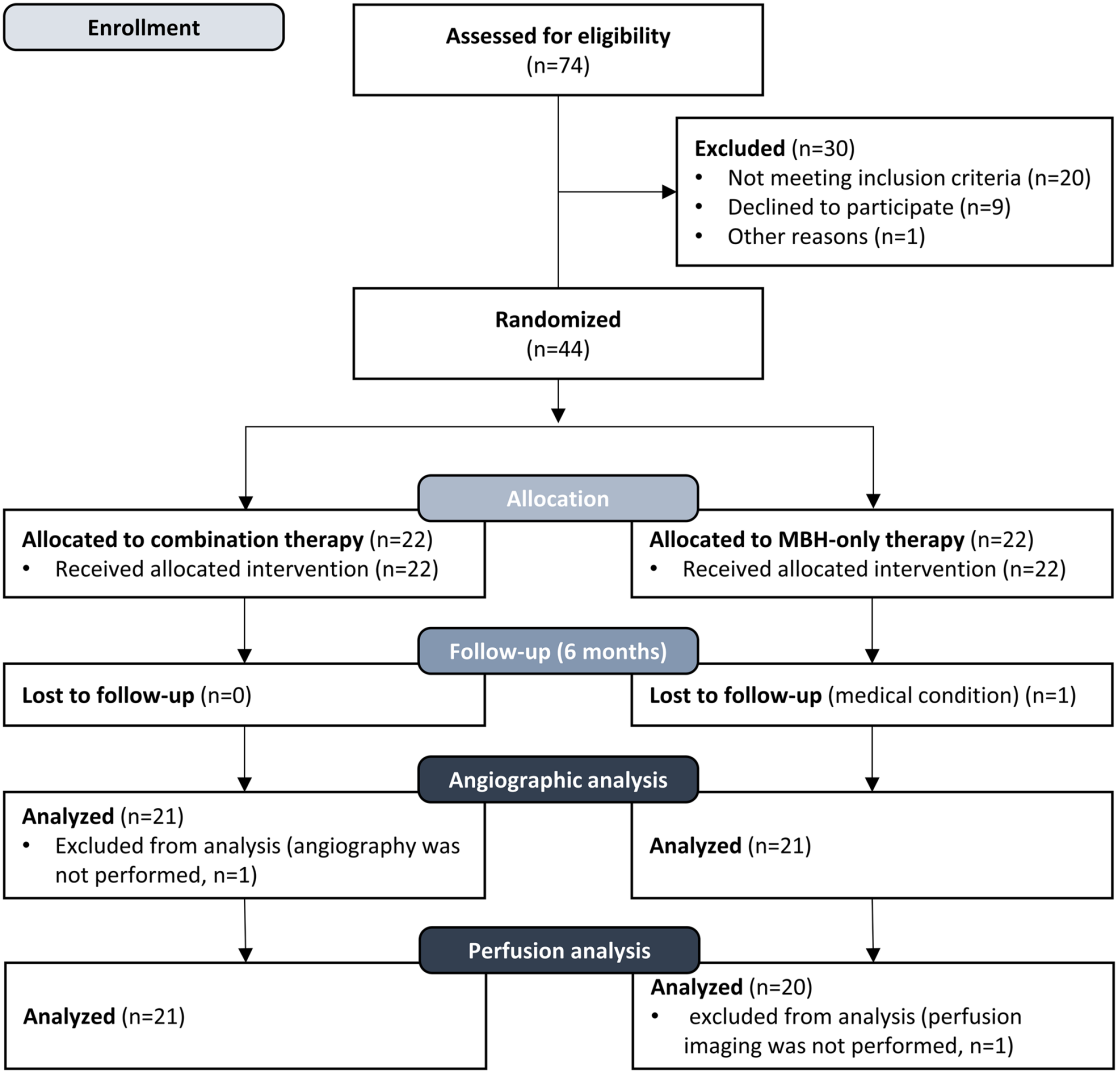
**Patient flow diagram.** MBH indicates multiple burr hole.

The NIMBUS (Neovascularization Induced by Mechanical Barrier Disruption and Systemic Erythropoietin in Patients With Cerebral Perfusion Deficits) trial was a single-center, prospective, randomized, 2-arm, placebo-controlled, phase-II clinical study as a blinded-end point evaluation (PROBE) designed in the Republic of Korea. The EPO used for this trial was provided by Dong-A pharmathetics (Eporon; DONG-A pharmaceutics, Seoul, South Korea). This study aimed to examine the efficacy and safety of EPO for transdural revascularization in patients with acute stroke with perfusion deficits undergoing cranial MBH procedures under local anesthesia. Informed consent of the trial was obtained from patients or patients’ legal guardians before study enrollment. This study was approved by the institutional review board of Ajou University Hospital (AJIRB-MED-CT2-15-187). The current trial has been registered with ClinicalTrials.gov. The study flowchart is presented in Figure 1.

### Study Population

The inclusion criteria were as follows: (1) aged 20 to 85 years, (2) acute ischemic stroke (ischemic infarction on diffusion-weighted imaging confirmation or transient ischemic attack without radiographic evidence) within 14 days after symptom onset with definite neurological deficits characterized by an initial National Institutes of Health Stroke Scale (NIHSS) score of <20, (3) confirmation of an atherosclerotic or steno-occlusive stroke mechanism (proximal cerebral arteries) on CT angiography or magnetic resonance angiography, (4) stage II or III perfusion impairment (with at least a reduction in regional cerebral blood flow and overwhelmed compensatory vasodilator capacity where hemodynamic failure was associated with the highest stroke risk),^16^ and (5) provision of written informed consent.

The exclusion criteria were as follows: (1) primary intracerebral hemorrhage or parenchymal hemorrhagic transformation of infarction as defined in the European Cooperative Acute Stroke Study), subarachnoid hemorrhage, arteriovenous malformation, cerebral aneurysm, or cerebral neoplasm; (2) treatment with a thrombolytic agent <24 hours before enrollment; (3) score of ≥1 on the NIHSS item 1a; (4) prestroke modified Rankin Scale score of ≥2; (5) uncontrolled hypertension (irregular systolic blood pressure of >150 mm Hg; (6) previous treatment with EPO; (7) patients who had not undergone 4-vessel cerebral angiography; (8) screening laboratory abnormalities such as a hemoglobin level of >14 g/dL, prolonged prothrombin time or partial thromboplastin time, serum creatinine level of >2.0 mg/dL, blood urea nitrogen level >40, thrombocytopenia or neutropenia as defined by the lower limit of normal for the platelet count or white blood cell count, respectively (absolute neutrophil count of >1800/mm^3^ required for participation), or >2× the normal values on liver function tests (serum glutamic oxaloacetic transaminase, serum glutamic pyruvic transaminase, and total bilirubin levels).

### Management

After baseline evaluation, eligible patients were randomized in a 1:1 fashion using a computer-generated randomization procedure with a block size of 6, which blindly generated a statistician-directed random allocation into the EPO or placebo group for 3 consecutive days, followed by a 6-month follow-up period. All patients received acute stroke care, including stroke unit care, and flow augmentation therapy, including induced-hypertension therapy, as required. The combined therapy (cranial MBH procedure + intravenous EPO) was designed to promote revascularization. For the combined therapy, 33 000 U of EPO was intravenously injected over 3 consecutive days to a total of 100 000 U,^12^ and the MBH procedure was performed in an area of hemodynamic insufficiency under local anesthesia with 1% lidocaine injections.^9^ Patients in the MBH-only group (cranial MBH procedure + placebo) received an equivalent volume of saline and the same MBH procedure as that performed in the combined group. The patients were typically discharged after a 7-day observation period after the procedure. The revascularization status was accessed on 6-month transfemoral cerebral angiography (TFCA). The results of TFCA were blindly reviewed by experienced neuroradiologists who were not involved in the study.

### Outcome Measurements

The primary outcome was the presence of successful arteriogenesis appearing as hemispheric transdural revascularization. Local revascularization was also assessed as trans-hemispheric (any arteriogenesis and vascular filling via burr holes in each hemisphere) and trans-burr hole (any arteriogenesis and vascular filling in each burr hole) arteriogenesis. Hemispheric revascularization was assessed on the basis of the relative revascularization area (RA). Revascularization success was evaluated on the 6-month angiography lateral images. The relative RA was calculated as the RA/supratentorial area and graded as excellent (≥66%), good (≥33%), fair (<33%), and poor (0%) by a consensus of independent authors (Drs Lee and Lee). Computed tomographic perfusion values were automatically analyzed using brain perfusion software (syngo Volume Perfusion CT Neuro, Siemens Healthcare, Erlangen, Germany).

The secondary outcomes of adverse events were classified into (1) index-disease associated: fluctuation or early neurological deterioration, lesion extension, intrainfarct hemorrhagic transformation, or intrainfarct edema; (2) procedure-related neurological complications: any brain hemorrhage or subdural hygroma; (3) other procedure-related findings: fever, headache, or eyelid swelling; (4) recurrence of infarction during follow-up; and (5) other systemic complications during follow-up. The stroke severity was serially measured by NIHSS. Neurological fluctuation or deterioration was defined as an increase in the NIHSS of 2 or more during admission. The presence or absence of procedure-related neurological and medical complications were evaluated within 2 weeks of combination therapy. Any complications after procedure were documented from 2 weeks up to 6 months. Preprocedural modified Rankin Scale and postprocedural modified Rankin Scale at 6 months were measured. All medical and neurological complications were recorded if they occurred during the therapeutic period. All patients received standard stroke care in a comprehensive stroke unit until they became stable neurologically.

### Serological Markers Associated With Sufficient Revascularization

The baseline serum levels of MMP (matrix metalloproteinase)-2, MMP-9, VEGF (vascular endothelial growth factor), granulocyte colony stimulating factor, and interleukin 6 were determined using ELISA kits (R&D Systems, Minneapolis, MN) according to the manufacturer’s instructions. All assays were performed by a researcher who was blinded to the clinical data. The concentrations of the analytes in the samples were extrapolated from a standard curve by using a 4-parameter logistic curve fit in Gen5 software (BioTek Instruments, Winooski, VT). The mean levels of serological markers according to the presence of successful revascularization and relative RA grading were evaluated.

The neutrophil-to-lymphocyte ratio, platelet-to-lymphocyte ratio, and lymphocyte-to-monocyte ratio determined in the complete blood count (CBC) were used as markers of subclinical inflammation. They were calculated by dividing the number of neutrophils by the number of lymphocytes, the number of platelets by the number of lymphocytes, and the number of lymphocytes by the number of monocytes, respectively. The mean levels of CBC-based inflammation markers in relation to the presence of successful revascularization and relative RA grades were evaluated at the following time points: at baseline, after the procedure, and at 6 months.

### Sample Size Estimates

The primary hypothesis of this study was that the patients receiving EPO before undergoing cranial MBH procedures would exhibit higher rates of successful revascularization at 6 months on TFCA than would those receiving the placebo. The expected proportions of successful revascularization were 60% and 90% in the placebo and EPO groups, respectively, according to a previous cranial burr hole study in patients with moyamoya disease.^9^ We selected a target sample size of 18 participants per group to provide 80% power using the *Z* test with pooled variance, at a 2-sided α of 0.05 to prove a treatment effect. The total sample size was determined to be 44 hemispheres (patients) considering a 15% dropout rate for the primary outcome.

### Statistical Analysis

Continuous variables are presented as the means±SDs or medians (interquartile ranges), and categorical variables are presented as absolute and relative frequencies. To test for statistical significance between the groups, the χ^2^ or Fisher exact tests were used for categorical variables, while an independent *t* test and the Mann–Whitney *U* test were used for numerical variables.

For the analysis of hemispheric profiles, sufficient revascularization was classified as a relative RA of ≥33%. In relation to patient profiles, sufficient revascularization was classified as a relative RA of ≥33% in the unilateral population and a relative RA of ≥33% in any of the 2 hemispheres in the bilateral population. The clinical and radiological profiles were compared according to the 6-month revascularization status. All potential predictors of successful revascularization (trans-hemisphere revascularization) were entered into a univariate logistic regression model, including demographic variables (ie, age, sex, RNF213 c.14576G>A variant,^17^ and risk factors for stroke) and the use of EPO. Potential factors that were nonsignificant (p>0.2; IBM SPSS Statistics 22; IBM Corp., Armonk, NY) in the univariate analysis were sequentially deleted from the full multivariable model. The results are presented as odds ratios, as estimates of the relative risk with 95% CIs. Statistical significance was set at *P*<0.05.

### Standard Protocol Approvals, Registrations, and Patient Consents

The study was conducted according to the Declaration of Helsinki, and the protocol was approved by the Institutional Review Board of Ajou University Medical Center (AJIRB-MED-CT2-15-187). Medical consent was obtained before study enrollment directly from patients who could communicate or from their caregivers. This study is registered with ClinicalTrials.gov.

## Results

### General Demographics

During the study period, that is, between July 15, 2016, and July 16, 2019, we screened 74 patients. A total of 44 patients were allocated to the cranial MBH-only or cranial MBH and EPO (combined) groups, of which 2 patients were lost to follow-up. The remaining 42 patients (n=21 in each group) were included in the final analysis.

The 2 groups did not differ in baseline demographic characteristics (age, sex disparity, proportion of patients with moyamoya disease, incidence of the RNF 213 c.14576G>A variant, stroke risk factors, symptom-to-admission time, admission NIHSS score, other initial laboratory data) or clinical manifestations (symptom-to-burr hole procedure time, NIHSS score at discharge, modified Rankin Scale score at discharge, other laboratory data) in the post-MBH period. The detailed clinical profiles are described in Table 1.

**Table 1.**
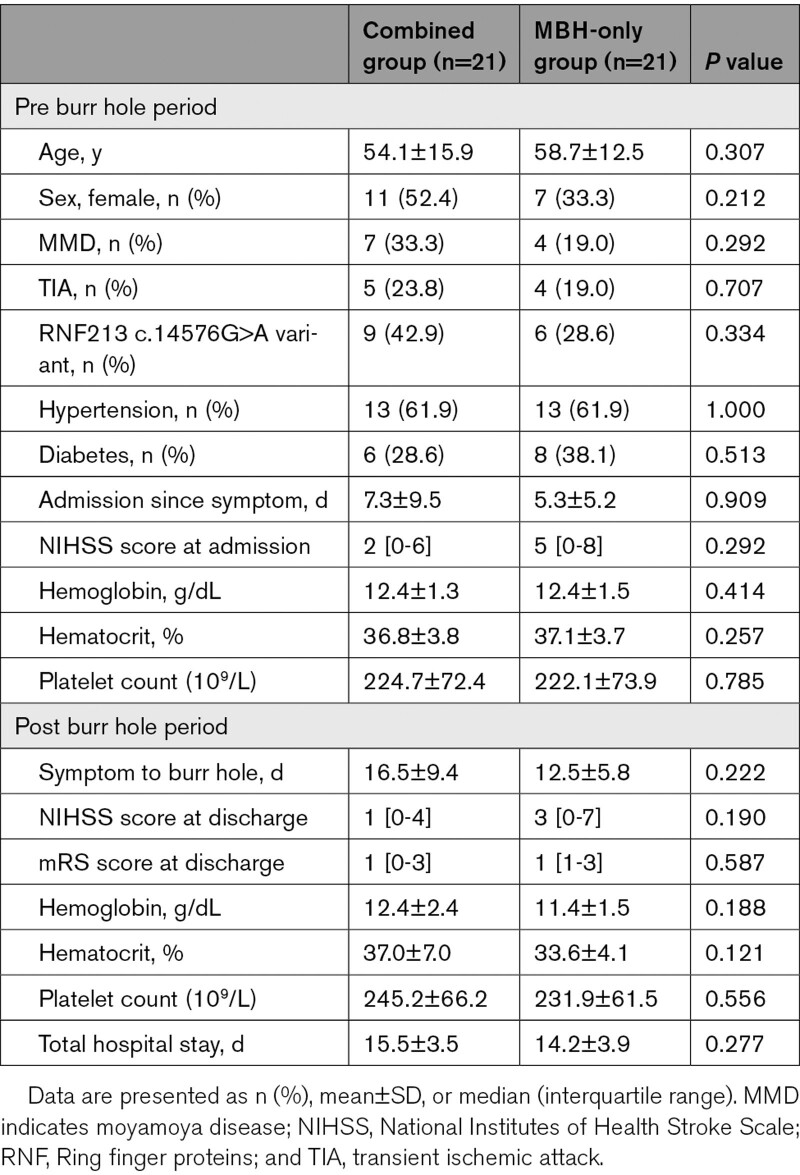
Clinical Profiles According to Treatment Groups

### Outcome Analyses

Successful transdural revascularization as the primary outcome was evaluated using blinded TFCA. The combined group exhibited higher rates of successful hemispheric revascularization (19/21 [90.5%] versus 12/21 [57.1%], *P*=0.014) and trans-burr hole revascularization (42/58 [72.4%] versus 30/58 [51.7%], *P*=0.001) than the MBH-only group. The combined group also showed significantly better revascularization in terms of the relative RA than the MBH-only group (*P*=0.037).

The secondary outcome profiles included clinical outcomes and CT perfusion parameters. The 2 groups did not differ in clinical outcomes and baseline CT perfusion parameters. However, compared with the MBH-only group, the combined group showed significant improvements in cerebral blood flow (*P*=0.034) and the mean transit time (*P*=0.008) at 6 months. The detailed outcome analyses are described in Table 2.

**Table 2.**
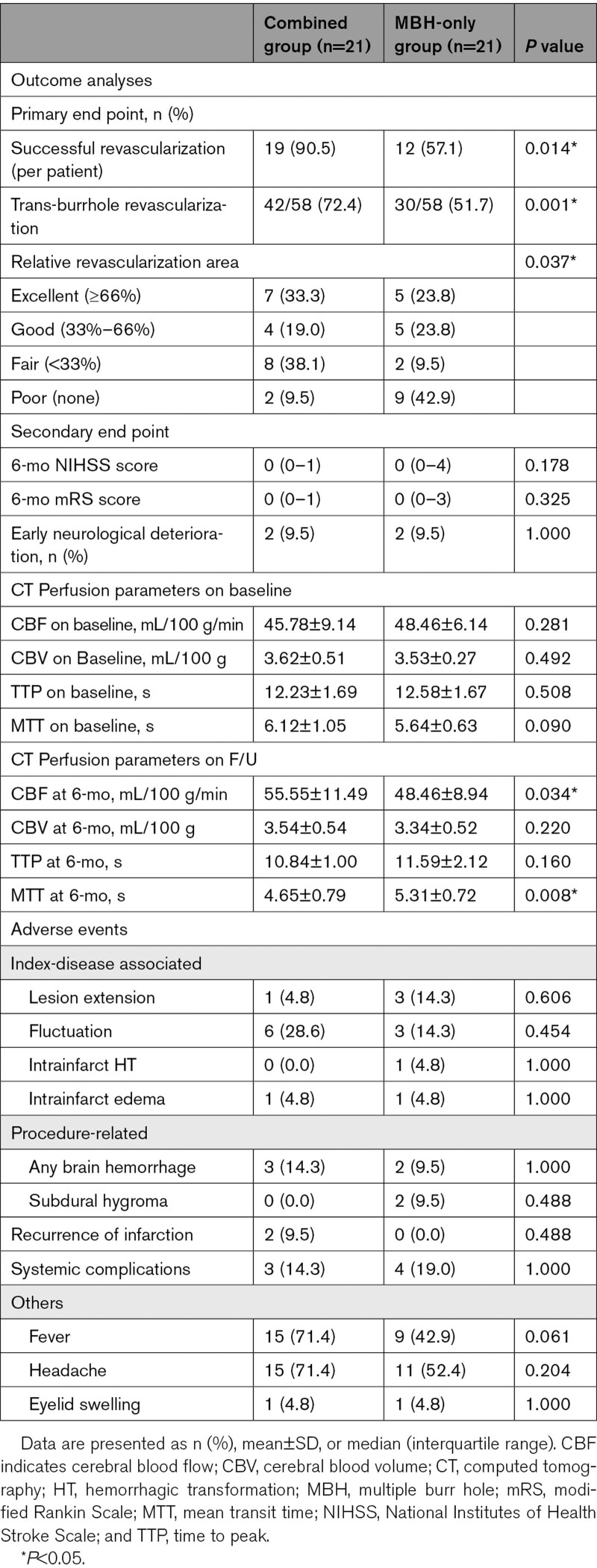
Outcome Analyses and Adverse Events

**Figure 2.**
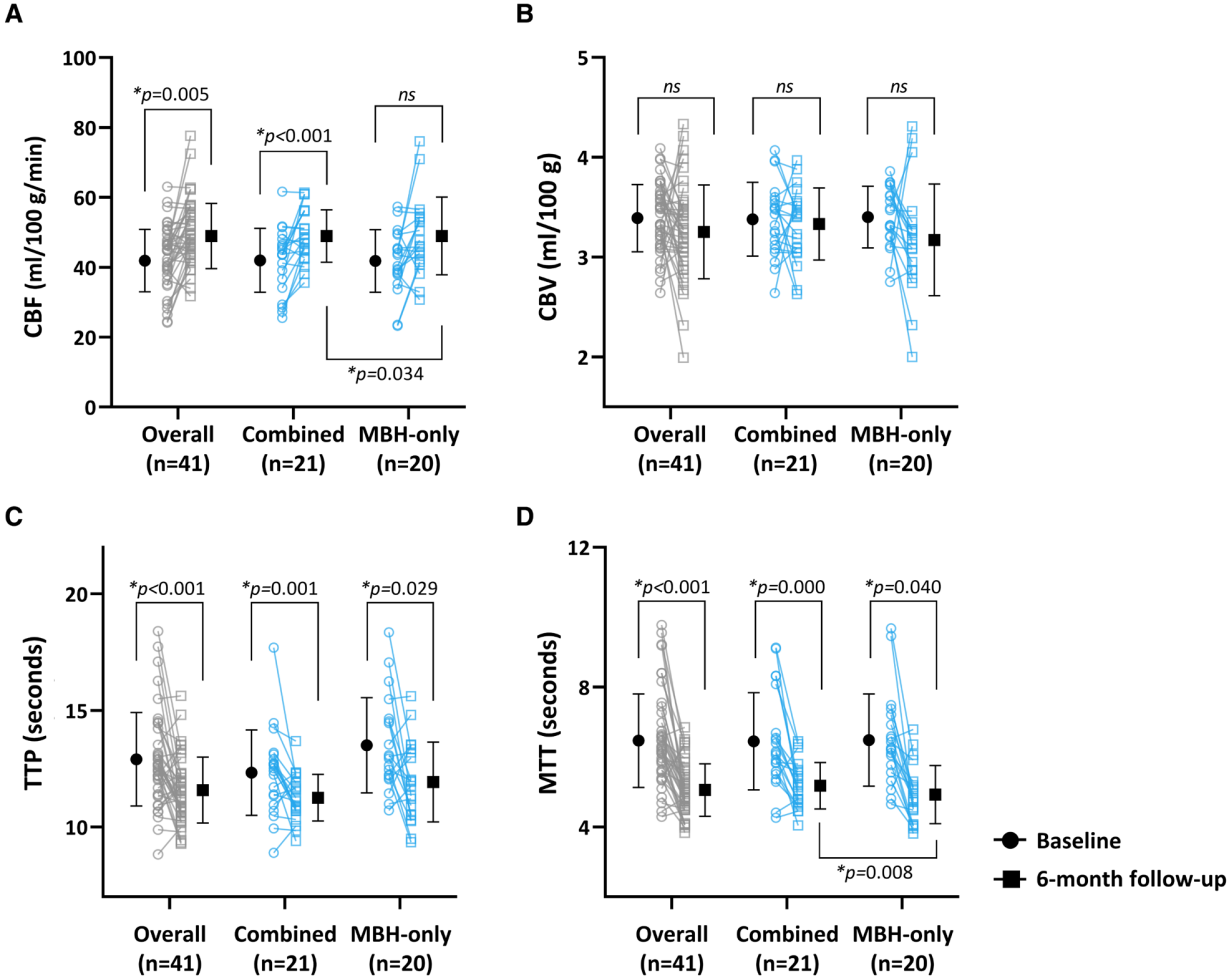
**Sequential changes in perfusion parameters at baseline and 6-mo follow-up.** The mean cerebral blood flow (CBF), cerebral blood volume (CBV), time-to-peak (TTP), and mean transit time (MTT) at baseline (black circle), and 6-mo follow-up (black square) were sequentially plotted for the overall study population (gray symbol) and the 2 treatment groups (blue symbol). **A**, The CBF values significantly increased in the overall (*P*=0.005) and combined groups (*P*<0.001). **B**, No changes were observed in the CBV values of all groups. **C**, The TTP values significantly decreased in all groups (overall, *P*<0.001; combined, *P*=0.001; multiple burr hole [MBH]-only, *P*=0.029). **D**, The MTT values significantly decreased in all groups (overall, *P*<0.001; combined, *P*=0.000; MBH-only, *P*=0.040). Data are presented as the mean±SD.

Figure 2 illustrates the changes in perfusion parameters. Compared with baseline, a significant increase in cerebral blood flow (*P*<0.001) and significant improvements in the time maps, including the time-to-peak (*P*=0.001) and mean transit time (*P*<0.001), were observed in the combined group at the 6-month evaluation. However, while the MBH-only group exhibited a significant increase in the time maps, including the time-to-peak (*P*=0.029) and mean transit time (*P*=0.040), significant changes in the cerebral blood volume and cerebral blood flow were not found.

### Adverse Events

No intergroup differences were observed in adverse events such as (1) index-disease associated complications, including fluctuation or early neurological deterioration, lesion extension, intrainfarct hemorrhagic transformation, and intrainfarct edema; (2) procedure-related neurological complications, including any brain hemorrhage or subdural hygroma; (3) other procedure-related findings, including fever, headache, and eyelid swelling; (4) recurrence of infarction during follow-up; and (5) other systemic complications during follow-up. The detailed adverse events are described in Table 2.

### Clinical Predictors of Successful Revascularization

In the univariate analysis, female sex, presence of the RNF213 c.14576G>A variant, and the use of EPO were associated with successful revascularization. Even after adjustment for potential determinants, the use of EPO (odds ratio, 6.41 [95%CI, 1.08–38.02]; *P*=0.041) was an independent predictor of successful hemispheric revascularization in the multivariate analysis (Table 3).

**Table 3.**
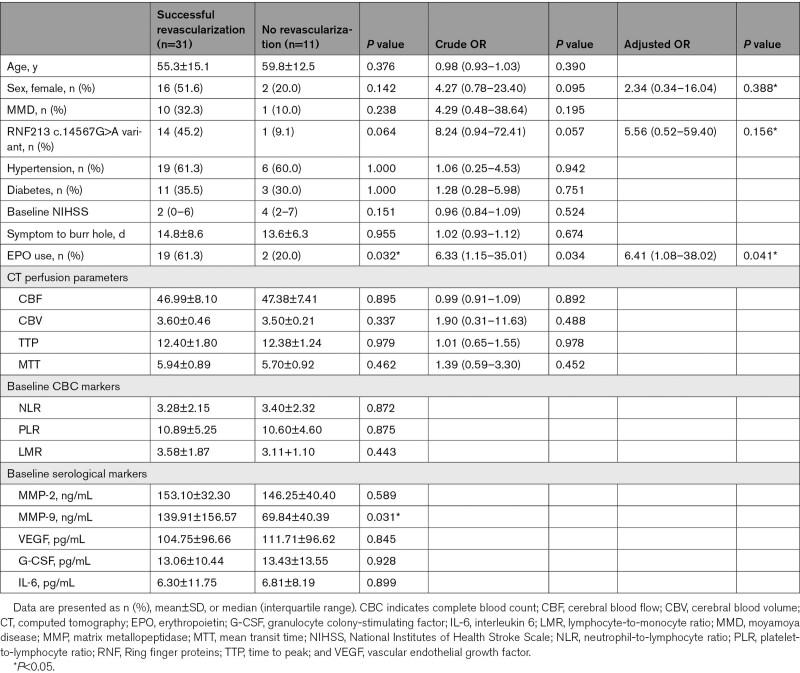
Clinical Characteristics According to Successful Revascularization

### Serological Markers of Sufficient Revascularization

To investigate the serological markers of sufficient revascularization (presence of any transdural revascularization), the baseline values of sequential CBC markers and potential biomarkers were compared between the successful revascularization and the no-revascularization groups. The 2 groups exhibited no differences in CBC markers at baseline, after the procedure, and at the 6-month follow-up measurement. Further, no significant differences in serological markers, with the exception of a significant increase in the baseline MMP-9 level (139.9±156.6 versus 69.8±40.4 ng/mL, *P*=0.031) were noted in the successful revascularization group.

## Discussion

**Figure 3.**
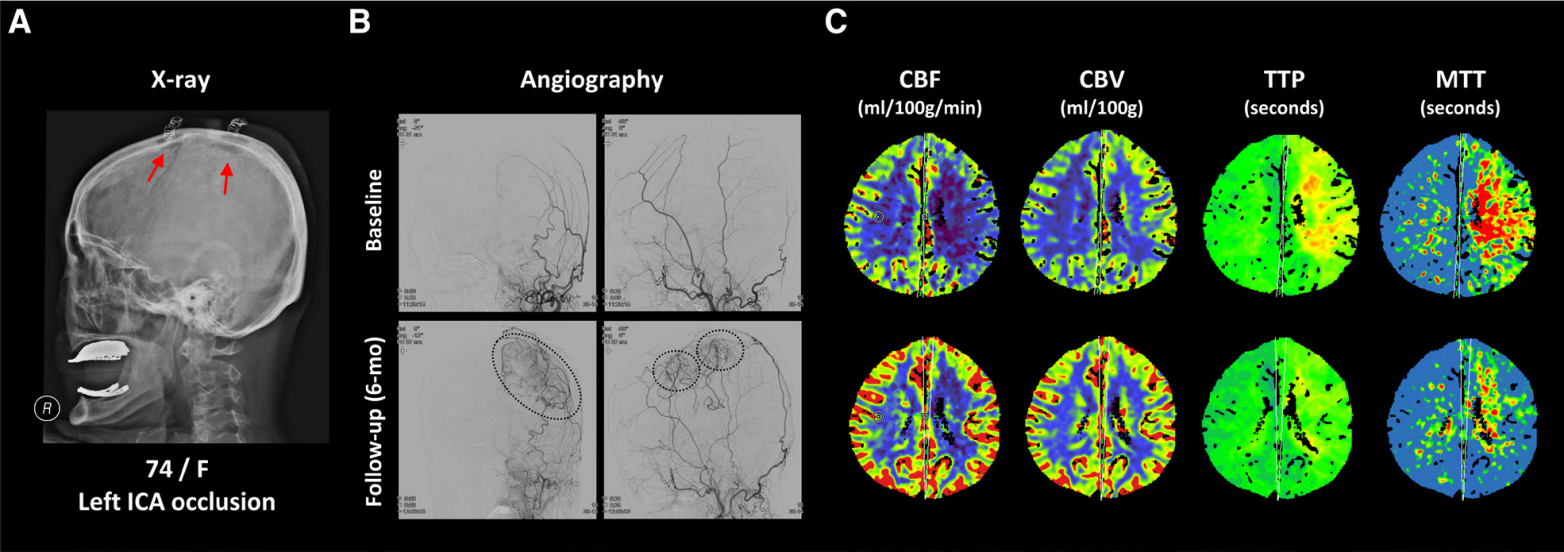
**Representative case showing good transdural collaterals after combination therapy. A**, Skull radiography after combination therapy in a 74-year-old woman with left internal carotid artery occlusion. Red arrows indicate the multiple burr hole (MBH) region. **B**, Angiography and (**C**) perfusion CT (cerebral blood flow [CBF], cerebral blood volume [CBV], time-to-peak [TTP], and mean transit time [MTT]) at baseline and 6 mo after combination therapy. The dotted ellipses indicate the transdurally developed arteriogenesis in the MBH regions.

This trial aimed to compare the efficacy and safety of cranial MBH procedures with or without intravenous EPO pretreatment for transdural revascularization in patients with acute symptomatic stroke. Our results revealed a significant improvement in hemispheric perfusion parameters and successful transdural revascularization at the 6-month follow-up in a considerable number of patients (Figure 3). Compared with the MBH-only group, the combined (MBH + EPO) group showed a significantly greater incidence of successful trans-hemisphere revascularization and trans-burr hole arteriogenesis. Considering the vulnerability of these acute patients to surgical complications, the incidence of adverse events was also found to be limited to acceptable rates during and after the procedure. After adjustment for all potential confounding factors, the use of EPO was found to be an independent predictor of successful transdural revascularization, suggesting that this combination therapy could be applied to the acute setting of ischemic stroke with perfusion impairment.

### MBH-Based Revascularization Strategy in Acute Ischemic Stroke

Our findings indicate that the cranial MBH-based strategy was associated with a successful revascularization rate of ≥57% and acceptable rates of complications during and after such procedures. In several studies, cranial MBH procedures involving the extracranial vascular system have been employed to treat progressive cerebrovascular occlusive disorders such as moyamoya disease with insufficient intracranial blood flow.^2,6,8^ However, these procedures have been applied to a narrow range of situations with chronic cerebral perfusion impairment excluding acute settings. This method of revascularization is relatively safer than other bypass surgeries that may be associated with postoperative complications.^4,5^ Theoretically, MBH procedures lead to temporary wound injury and repair due to mechanical barrier disruption of the brain’s protective layers between the intracranial and extracranial carotid systems, which is caused by a combination of the cranial burr hole and minor disruptions of the meninges.^9,18^ However, these procedures cannot guarantee stable new vessel formation via the transdural collaterals from the enriched extracranial environment.^6,8^

### Revascularization Augmentation Using MBH Procedures With EPO Pretreatment

In our trial, the combined (MBH + EPO) group exhibited significantly better trans-hemispheric revascularization and trans-burr hole arteriogenesis than did the MBH-only group. Several experiments have demonstrated that EPO contributes to angiogenesis, showing an angiogenic potential similar to that of VEGF.^19^ Previous studies of stroke in animals and humans have also indicated that EPO induces neuroprotective effects involving reductions in oxidative stress, apoptosis, and inflammation.^10,19–23^ Previous studies of stroke in animals and humans have also indicated that EPO induces neuroprotective effects involving reductions in oxidative stress, apoptosis, and inflammation.^10,19–23^ Moreover, recent animal and human studies showed that EPO facilitates angiogenesis after cranial MBH procedures, suggesting that it can induce angiogenesis and arteriogenesis even in acute stroke settings.^9,18^ Therefore, the findings of the current study are consistent with the previous findings showing that MBH procedures and EPO can promote vasculogenesis even in acute environments.

### Biomarkers of Augmented Revascularization

Serological markers of revascularization can provide additional physiological insights into the facilitation of transdural revascularization by a combined (MBH + EPO) therapeutic method in patients with acute stroke with perfusion impairment. While hypoxia in normal tissues promotes regional revascularization, progressive intracranial stenosis prevents it, forming a poor intracranial milieu. In contrast, a healthy extracranial milieu can be augmented of its arteriogenic potency by increased shear stress and circulating cytokines.^18^ EPO pretreatment can augment arteriogenic potency, while a simple cranial MBH procedure breaks the barrier between the 2 milieus. The mechanical gradient of blood pressure can initiate backward vessel sprouting from the quiescent phalanx of the extracranial endothelium, while circulating cytokines form a chemotactic gradient.^24–26^ Eventually, the successfully directed transdural collaterals replace the poor internal carotid flow. Analyses of baseline serological biomarkers before the MBH procedure support this process. Several studies have reported that MMPs play an important role in vascular tissue remodeling during stroke recovery.^27–29^ Furthermore, our data suggest that the increase in MMP-9 levels in the patients with successful revascularization is associated with previous findings that increased the level of MMP-9 as an important angiogenic factor in patients with moyamoya disease.^30,31^ Therefore, we believe that a higher level of MMP-9 is observed in cases showing sufficient revascularization, which is triggered by the initial hypoxic insult. We used baseline and sequential CBC markers to evaluate the systemic inflammation status. However, there was no difference between the 2 groups. This indirectly suggests that MBH has a negligible effect on systemic inflammation, unlike other extensive neurosurgery operations.

### Limitations

The current study had several strengths and limitations. Since all of the patients in the current study were randomly registered, the findings have now clarified the positive revascularization effects of combined MBH and EPO therapy. Despite the strengths of the study, further randomized controlled trials using standard MBH protocols are needed in the future because it remains difficult to simplify the MBH procedure, which prevented the inclusion of multiple institutions in the NIMBUS trial. Since this is a small study that relies on positive EPO treatment effects derived from statistically significant results, the interpretation may be biased even for minor baseline differences. Favorable outcomes in the current study may contribute to potential clinical benefits in further phase III clinical trials with appropriate sample size. We also cannot clarify the exact timing of arteriogenesis. While it is important for such verifications to optimize the indications for the combined therapy, the invasive nature of TFCA limits evaluation of the success of transdural revascularization. Thus, it is necessary to develop a noninvasive technique to document transdural revascularization in future studies.

### Conclusions

Combined therapy is safe and feasible for successful transdural revascularization in patients with acute steno-occlusion with perfusion impairment.

## Article Information

### Acknowledgments

Dr Hong wrote the article and was involved in the conception and design of this study, interpretation of data, and critical revision for important intellectual content. Drs Choi and Park wrote the draft and acquired, analyzed, and interpreted the data. H.S. Shin interpreted the data. Drs Lee and Lee acquired the data. Dr Lim was involved in the conception and acquisition of data.

### Sources of Funding

This research was supported by a grant of the Korea Health Technology R&D Project through the Korea Health Industry Development Institute (KHIDI), funded by the Ministry of Health & Welfare, Republic of Korea (grant number: HR21C1003).

### Disclosures

None.

## Supplementary Material


