# Supplementary material for: Transdural Revascularization by Multiple Burrhole After Erythropoietin in Stroke Patients With Cerebral Hypoperfusion: A Randomized Controlled Trial
Source: Stroke. 2022 May 17;53(9):2739–48. doi: 10.1161/STROKEAHA.122.038650 (PMC9389942; doi:10.1161/STROKEAHA.122.038650)
Supplement: Supplementary file 2 [file str-53-2739-s002.pdf]

* **CONSORT Reporting Guidelines**

This study is reporting on a clinical randomized control trial:

Yes

Clinical trial reports should comply with the Consolidated Standards of Reporting Trials (CONSORT) including its additional extensions as appropriate. This should include a flow diagram presenting the screening, enrollment, intervention allocation, follow-up, and data analysis with number of subjects for each. Please refer specifically to the CONSORT Checklist of items to include when reporting a randomized clinical trial and provide the completed checklist at the time of submission.

Please confirm and provide the following details at this time:

Clinical Trial Registration

Clinical trials should be registered in an appropriate online trial registry at or before the onset of participant enrollment. In the box below, please provide the name of the trial registry, the registry URL, and the trial registration number. If your study is a clinical trial but not registered, please provide an explanation below.

https://clinicaltrials.gov/ct2/show/NCT02603406

Sex and Race/Ethnicity Specific Results

Sex and race/ethnicity specific results of the trial's primary outcomes are reported regardless of whether there are significant differences by sex or race.

Yes

Diverse Representation - Participants

Were steps taken to ensure diverse representation among trial participants, and if so, are these efforts outlined in the methods? If No, a lack of diversity should be explained and listed as a limitation.

Yes

Diverse Representation - Steering Committee

Were steps taken to ensure diverse representation among the trial steering committee, and if so, are these efforts outlined in the Methods including demographic information? If No, a lack of diversity should be explained and listed as a limitation.

Yes

Date completed: 04/14/2022 02:22:02

User pid: 6841
